# Novel Biomarkers for Prognostic Assessment of Patients with Acute Exacerbation of COPD in the Emergency Department—Tools to Enhance the Quality of Care in Critical Patient Management

**DOI:** 10.3390/diagnostics16010122

**Published:** 2026-01-01

**Authors:** Raluca Mihaela Tat, Sonia Luka, Eugenia Maria Lupan-Mureșan, George Teo Voicescu, Luca David, Adela Golea, Ștefan Cristian Vesa

**Affiliations:** 1 Cluj-Napoca County Emergency Clinical Hospital, 3-5 Clinicilor Street, 400347 Cluj-Napoca, Romania; raluca.tat@umfcluj.ro (R.M.T.); sonia.luka@umfcluj.ro (S.L.); muresan.eugenia@umfcluj.ro (E.M.L.-M.); george.voicescu@uniupo.it (G.T.V.); 2 Department 6 Surgery, Emergency Medicine Discipline, “Iuliu-Hatieganu” University of Medicine and Pharmacy, 8 Victor Babes Street, 400347 Cluj-Napoca, Romania; 3 CRIMEDIM—Center for Research and Training in Disaster Medicine, Humanitarian Aid and Global Health, Università del Piemonte Orientale, 28100 Novara, Italy; 4 Faculty of Medicine, “Iuliu-Hatieganu” University of Medicine and Pharmacy, 8 Victor Babes Street, 400347 Cluj-Napoca, Romania; david.luca@elearn.umfcluj.ro; 5 Cluj-Napoca Clinical Hospital of Infectious Diseases, 23 Iuliu Moldovan Street, 400000 Cluj-Napoca, Romania; stefan.vesa@umfcluj.ro; 6 Department 1 Functional Sciences, Discipline of Pharmacology, Toxicology and Clinical Pharmacology, Faculty of Medicine, Iuliu-Hatieganu University of Medicine and Pharmacy, 23 Marinescu Street, 400337 Cluj-Napoca, Romania

**Keywords:** COPD, emergency department, prognostic score, resistin, CC16, S100β, IL-6, TNF-α

## Abstract

**Background/Objectives**: Chronic obstructive pulmonary disease (COPD) remains a major global health problem, affecting over 300 million people worldwide. Its high morbidity and mortality rates impose substantial psychosocial and financial burdens on patients and healthcare systems. In the emergency setting, managing acute exacerbations of COPD (AECOPD) poses a major clinical challenge, as these patients often present with multi-organ dysfunction secondary to hypoxia and hypercapnia. Identifying reliable prognostic biomarkers could improve early risk stratification, guide therapeutic decisions, and enhance patient outcomes. **Methods**: This multicenter, prospective, observational study aims to evaluate the prognostic significance of several novel biomarkers—resistin, club cell secretory protein 16 (CC16), interleukin-6 (IL-6), tumor necrosis factor-alpha (TNF-α), S100β protein—alongside conventional markers such as N-terminal-pro–B-type-Natriuretic-Peptide (NT-proBNP), D-dimer, high-sensitivity troponin I (hs-cTnI), C-reactive protein (CRP), and procalcitonin in patients with AECOPD admitted to the Emergency Department (ED). Blood samples will be collected at admission. The novel biomarkers (resistin, CC16, IL-6, TNF-α, S100β) will be measured using standardized ELISA kits, while conventional biomarkers (NT-proBNP, troponin I, CRP, procalcitonin) will be analyzed using routine automated clinical laboratory methods. Correlations between biomarker levels, clinical and imaging data, severity scores (GCS, SOFA, CFS, Ottawa COPD Risk Scale, DECAF, BAP-65), and short-term outcomes (hospital discharge status and 28-day survival) will be assessed. The study has received approval from the Ethics Committee of the “Iuliu-Hatieganu” University of Medicine and Pharmacy, Cluj-Napoca, and all participating hospitals. Written informed consent will be obtained from all participants or their legal representatives. **Results:** This study protocol does not report results, as data collection and analysis are ongoing. **Conclusions /Expected Impact:** By identifying novel biomarkers with prognostic and pathophysiological relevance, this research aims to inform the development of early risk stratification tools and support future evidence-based approaches to the management of critically ill COPD patients in the ED.

## 1. Introduction

Chronic obstructive pulmonary disease (COPD) represents one of the leading causes of morbidity and mortality worldwide, accounting for substantial healthcare expenditures and social burden. Despite advances in preventive and therapeutic strategies, COPD exacerbations remain a major cause of emergency department (ED) admissions and hospitalizations [1,2]. According to the 2023 Global Initiative for Chronic Obstructive Lung Disease (GOLD) report, COPD is now recognized as a complex, systemic condition extending beyond the respiratory tract, involving inflammatory and metabolic alterations that contribute to multi-organ dysfunction [3].

Acute exacerbations of COPD (AECOPD) are pivotal events in disease progression, often precipitating irreversible declines in pulmonary function, worsening quality of life, and escalating mortality risk. During exacerbations, the systemic inflammatory response becomes amplified, leading to acute respiratory failure and subsequent cardiovascular or neurological complications. Patients admitted to the ED frequently present with hypoxia, hypercapnia, and hemodynamic instability, which may evolve into multi-organ dysfunction. The accurate early assessment of prognosis in this setting remains challenging, as current risk stratification tools, although widely used, including the Modified Medical Research Council (mMRC) scale, the Sequential Organ Failure Assessment (SOFA) score, the Glasgow Coma Scale (GCS), the DECAF score for in-hospital mortality, the BAP-65 score, the Ottawa COPD Risk Scale, and the Clinical Frailty Scale (CFS), do not incorporate objective biological markers of systemic injury or repair [4,5,6,7,8,9].

Recent advances in molecular diagnostics have underscored the potential of circulating biomarkers to reflect the underlying pathophysiological processes of COPD. Several molecules involved in systemic inflammation, oxidative stress, and epithelial injury have emerged as candidates for prognosis and therapeutic guidance. Among these, resistin, club cell secretory protein (CC16), interleukin-6 (IL-6), tumor necrosis factor-alpha (TNF-α), and S100β protein stand out for their plausible mechanistic roles in inflammatory cascades and tissue injury, yet their clinical value in AECOPD remains insufficiently characterized [4,5,6].

Resistin, an adipokine and proinflammatory cytokine, has been linked to endothelial dysfunction, cardiovascular injury, and sepsis severity [10,11]. Elevated resistin levels correlate with the extent of multi-organ failure and short-term mortality in critical illness, suggesting a potential role in predicting poor outcomes in COPD exacerbations where systemic inflammation predominates [4,5,10,11].

CC16, is produced by non-ciliated bronchiolar epithelial cells and functions as an anti-inflammatory and epithelial-protective factor [6,12]. Decreased CC16 concentrations have been associated with epithelial barrier disruption, pulmonary hyperpermeability, and increased susceptibility to airway injury. In COPD, CC16 deficiency correlates with accelerated disease progression and poorer lung function indices, making it a potential marker of respiratory epithelial integrity [6,12].

IL-6 and tumor necrosis factor-alpha TNF-α are well-established mediators of systemic inflammation and have been consistently associated with disease severity and exacerbation frequency in COPD [13,14,15]. Both cytokines drive neutrophilic inflammation and contribute to skeletal muscle wasting, oxidative stress, and vascular dysfunction. Elevated IL-6 and TNF-α levels at exacerbation onset have been shown to predict adverse clinical outcomes and increased mortality, underscoring their potential role in biomarker-based prognostic models [13,14,15].

S100β protein, a calcium-binding glial marker, reflects astrocytic activation and neuronal injury. Although traditionally studied in acute brain injury and cardiac arrest survivors [16,17], S100β may also indicate cerebral hypoxia secondary to respiratory failure in severe COPD exacerbations. Elevated serum levels could thus signify subclinical neurological impairment, contributing to poor recovery and increased mortality in critical COPD cases [16,17].

Despite this growing body of evidence, no comprehensive study has yet examined the combined prognostic value of these biomarkers in AECOPD patients presenting to the Emergency Department. Integrating inflammatory, epithelial, and neuroinjury markers into a single prognostic framework could bridge the existing gap between clinical assessment and pathophysiological reality, providing a more nuanced understanding of disease severity and recovery potential.

The proposed study seeks to validate the prognostic significance of resistin, CC16, IL-6, TNF-α, and S100β in a real-world emergency care population. By correlating their serum concentrations with established clinical scores, imaging parameters, and short-term outcomes, this research aims to identify biomarker thresholds predictive of mortality and ventilator support escalation. Ultimately, these findings could support the development of a biomarker-based early prognostic score, offering a precision-medicine approach to COPD management in the ED (Table 1). Specifically, we will evaluate whether baseline biomarker concentrations obtained at ED admission correlate with the subsequent level of respiratory support required during hospitalization, supplemental oxygen, non-invasive ventilation (NIV), or invasive mechanical ventilation (IMV), without influencing therapeutic decisions in real time.

## 2. Materials and Methods

### 2.1. Study Design and Setting

This research is designed as a prospective, analytical, longitudinal, multicenter observational study conducted by an institutional consortium including the “Iuliu-Hatieganu” University of Medicine and Pharmacy Cluj-Napoca and affiliated hospitals: County Emergency Clinical Hospital Cluj-Napoca (primary site), Municipal Clinical Hospital Cluj-Napoca, Clinical Hospital of Infectious Diseases Cluj-Napoca, Leon Daniello Pneumology Hospital Cluj-Napoca, and “Dr. Constantin Papilian” Military Emergency Hospital Cluj-Napoca, all equipped with intensive care units for the admission of critically ill patients and dedicated wards for non-critical COPD patients.

### 2.2. Study Population

The target population consists of adult patients (≥ 50 years) with a previous diagnosis of COPD presenting to the ED with an acute exacerbation requiring evaluation and management. Acute exacerbations will be defined according to the Rome Proposal, which defines an acute exacerbation as an event characterized by worsening dyspnea and/or cough and sputum over ≤14 days, often accompanied by tachypnea and/or tachycardia, and associated with increased local and systemic inflammation and physiological deterioration [18,19]. Exacerbation severity will be classified at the point of care as mild, moderate, or severe according to the Rome Proposal severity classification, based on objective clinical, physiological, and inflammatory parameters assessed at ED presentation [18,19]. Patients enrolled in the study will have a prior diagnosis of COPD, with GOLD spirometric grade and ABE category assessment established according to current GOLD recommendations at the Pneumology Clinic. The GOLD spirometric grades (I–IV) classify the physiological severity of airflow obstruction and are defined based on post-bronchodilator forced expiratory volume in 1 s (FEV_1_) expressed as percent predicted. This spirometric grading remains a fundamental descriptor of disease severity and is essential for the diagnostic characterization of COPD. Category A includes patients with low symptom burden and low risk of exacerbations. Category B includes patients with increased symptoms but low risk of exacerbations. Category E includes patients at high exacerbation risk, irrespective of the level of symptoms [3] (Table 2).

Patients presenting with conditions that mimic an acute COPD exacerbation will be excluded from the study. These include acute heart failure, acute coronary syndromes, major arrhythmias, pulmonary embolism, pneumothorax, acute asthma exacerbation, non-pulmonary sepsis, upper airway obstruction, and respiratory infections in patients without previously documented COPD. Additional conditions that may interfere with respiratory function and therefore lead to exclusion from the study are detailed in Table 3. In patients presenting to the ED with a previously confirmed diagnosis of COPD, infections such as pneumonia, bronchitis, or viral illnesses will be considered valid triggers of an acute exacerbation [18,19].

### 2.3. Sample Size and Study Duration

The study is planned to include approximately 100 consecutive eligible patients admitted over a 15-month period. The sample size was estimated based on local patient volumes and previous annual ED statistics (over 600 COPD admissions per year), ensuring sufficient statistical power to detect correlations between biomarker levels and clinical outcomes. Baseline biomarker values will be analyzed exclusively for prognostic correlation with the later need for oxygen therapy, NIV or IMV, and will not be used to guide acute therapeutic escalation or de-escalation.

Based on an estimated annual number of approximately 600 COPD presentations to the ED, we anticipated enrolling around 100 eligible patients meeting the study inclusion criteria. In these participants, five biomarkers (resistin, CC16, IL-6, TNF-α, and S100β protein) will be measured and correlated with disease severity and short-term outcomes.

A post hoc power analysis performed using G*Power software (version 3.1.9.7) for a two-tailed Mann–Whitney test, assuming a large effect size (Cohen’s d = 0.8) and a Bonferroni-adjusted significance level (α = 0.01), indicated a statistical power (1 − β) of approximately 0.91–0.92. This suggests that a sample size of 100 participants could be adequate for detecting large and clinically relevant differences in biomarker levels between well-defined severity or outcome groups. In patients with acute exacerbations of COPD, Vassiliou et al. reported higher serum resistin levels during the acute phase as compared to the stable phase after clinical recovery (median at admission 19.44 ng/mL vs. 9.70 ng/mL at follow-up), indicating a large difference between clinically distinct states [4]. We will reevaluate the sample size after enrollment of 20 patients, and we plan to extend the study to other centers, if necessary.

### 2.4. Ethical Considerations

The study has received ethical approval from the Ethics Committee of “Iuliu-Hatieganu” University of Medicine and Pharmacy Cluj-Napoca (No. AVZ 71/20 March 2025) and from the Ethics Committee of Cluj-Napoca County Emergency Clinical Hospital (No. 9333/17 March 2025). The study will comply with the Declaration of Helsinki, the European Union General Data Protection Regulation, and national research ethics legislation. All participants (or their legal representatives) will provide written informed consent prior to enrollment.

### 2.5. Data Collection Procedures

#### 2.5.1. Therapeutic Protocol

All patients will receive therapy appropriate to the stage of COPD and to any acute comorbidities, in accordance with current clinical guidelines. Patients presenting with fever secondary to infectious processes will receive antipyretic therapy according to current standards of care, with the aim of reducing elevated body temperature and restoring normothermia. In critically ill patients with suspected sepsis or septic shock, intravenous administration of isotonic crystalloid fluids, as well as inotropic and vasoactive medications, will be initiated according to the Surviving Sepsis Campain recommendation, with the aim of maintaining a urine output ≥0.5 mL/kg/hour and a mean arterial pressure (MAP) ≥ 70 mmHg, ensuring adequate cerebral perfusion [20]. The choice of vasopressor medication will be at the discretion of the attending physician. Patients requiring non-invasive or invasive mechanical ventilation will receive appropriate ventilatory support as indicated by the attending physician. All patients will undergo routine laboratory testing, as well as specific investigations relevant to their underlying pathology or comorbidities, along with radiologic and imaging studies necessary for optimal case management, at the discretion of the treating physician. Therapeutic decisions will be individualized according to disease severity and patient-specific characteristics, following the current evidence-based guidelines for COPD exacerbations (GOLD), sepsis and septic shock (Surviving Sepsis Campaign), pneumonia, and other acute conditions identified at presentation [3,20,21].

#### 2.5.2. Recorded Data

Demographic data: age, sex, place of residence (urban or rural), living conditions (home or long-term care facility), past medical history, chronic medication, hospitalization within the previous 30 days, and classification as end-stage or palliative care prior to ED admission will be recorded in the patient monitoring form at admission.

Parameters evaluated during the study at ED admission: systolic and diastolic blood pressure (SBP/DBP), mean arterial pressure (MAP), core temperature (T°C), Glasgow Coma Scale (GCS) score, blood glucose, urine output, peripheral oxygen saturation (SpO_2_), end-tidal (Et) carbon dioxide (CO_2_)—EtCO_2_ and EtCO_2_ trend; oxygen therapy requirements [(mask type, O_2_ flow rate per minute, Fraction of Inspired Oxygen (FiO_2_)]; need for NIV with ventilatory parameters; need for IMV with ventilatory parameters, including airway resistance and lung compliance; requirement for vasoactive support; and medication administered in the ED.

Laboratory data collected at ED admission: routine laboratory investigations will be performed at ED admission and will include a complete blood count, serum creatinine, urea, liver enzymes [Alanine Aminotransferase (ALT) and Aspartate Aminotransferase (AST)], total bilirubin, blood glucose, and a standard coagulation profile. Arterial blood gas analysis will be conducted to assess potential of hydrogen (pH), lactate, bicarbonate (HCO_3_^−^), partial arterial pressures of oxygen (PaO_2_) and carbon dioxide (PaCO_2_), fraction of inspired oxygen (FiO_2_), the PaO_2_/FiO_2_ ratio, the end-tidal to arterial CO_2_ gradient (EtCO_2_–PaCO_2_), and the alveolar—arterial oxygen difference (A–aDO_2_). Additional analyses well comprise sepsis and infection biomarkers [C-reactive protein (CRP), procalcitonin, and fibrinogen], cardiac dysfunction biomarkers [high-sensitivity troponin I (hs-cTnI) and N-terminal-pro–B-type-Natriuretic-Peptide (NT-proBNP)], and the thrombosis marker D-dimer.

Imaging studies performed at ED admission: Imaging studies well be performed at ED admission according to clinical indications and will include chest radiography, chest computed tomography (CT), and lung ultrasound examinations. In selected cases, diaphragmatic motion and compliance will also be assessed by bedside ultrasonography to evaluate respiratory mechanics and ventilatory function.

#### 2.5.3. Biomarkers Selected for Assessment in the Study

Serum concentrations of resistin, CC16, IL-6, TNF-α and S100B protein will be measured using standardized sandwich ELISA assays kits (Elabscience^®^, Biotechnology Inc., Wuhan, Hubei, China). Blood samples will be collected at ED admission into 5 mL biochemistry vacutainers with gel separator. After collection, samples will be centrifuged, at 3500 rpm and the resulting serum will be either processed fresh within a maximum of eight hours or stored at −70 °C until analysis, according to the manufacturer’s recommendations. Hemolyzed or lipemic samples will be excluded.

All assays will be performed following the manufacturer’s protocol, including reagent preparation, incubation conditions, calibration curve generation, and internal quality control verification. Optical density will be measured at 450 nm, and biomarker concentrations will be calculated using a four-parameter logistic (4-PL) curve model.

The analytical characteristics of each ELISA assay, detection range, analytical sensitivity (LOD/LOQ), intra- and inter-assay variability (CV%), assay specificity and cross-reactivity, and reference intervals for normal adult populations, are summarized in Table 4.

#### 2.5.4. Data Monitoring

At ED admission, all patients will undergo assessment using validated severity and prognostic scores available on the MDCalc platform (www.mdcalc.com). The following tools will be applied: mMRC Dyspnea Scale for grading dyspnea severity, the SOFA score, GCS, for evaluating neurological impairment secondary to hypoxia or hypercapnia, and the CFS, adapted for triage levels used in Romania, to estimate frailty and predict mortality. Additional prognostic scores specific to COPD will be applied, including the Ottawa COPD Risk Scale (predicting 30-day mortality or severe adverse events such as myocardial infarction or the need for intubation), the DECAF Score for in-hospital mortality in acute exacerbations of COPD, and the BAP-65 Score for in-hospital mortality prediction.

Serum biomarker levels will be measured according to the previously described analytical protocol. Based on clinical status and therapeutic requirements, study participants will be classified into three groups: COPD patients requiring supplemental oxygen therapy, those requiring NIV, and those requiring IMV. These measurements are intended solely to establish prognostic associations with subsequent respiratory support requirements and will not influence real-time clinical management.

At discharge from the ED or before hospitalization in the Pulmonology Department or the Internal Medicine Department, patient outcomes will be documented as improved (reduction in dyspnea and clinical instability, no requirement for escalation of respiratory support, decreased level of respiratory support, and evidence of improving gas exchange), stable (no significant improvement but no deterioration, with the same level of respiratory support maintained), worsened (increased respiratory distress, need for escalation of respiratory support, and worsening gas exchange or hemodynamics), or deceased (ED mortality). The total duration of hospitalization will be recorded, as well as survival time, including the date of death if it occurred during the ED stay or hospitalization. Early survival will additionally be assessed at 28 days through telephone follow-up.

#### 2.5.5. Data Management and Confidentiality

Data will be entered into a secured electronic database accessible only to the research team. All patient identifiers will be removed and replaced with unique study codes. The database will be password-protected and stored on encrypted institutional servers.

#### 2.5.6. Statistical Analysis

All statistical analyses will be performed using appropriate parametric or non-parametric tests, depending on data distribution as assessed by the Shapiro–Wilk test. Descriptive statistics will be used to summarize baseline demographic, clinical, and laboratory characteristics, with results expressed as mean ± standard deviation (SD) or median (interquartile range, IQR) for continuous variables, and as absolute and relative frequencies for categorical variables. Associations between biomarker concentrations and clinical severity scores (SOFA, DECAF, Ottawa COPD Risk Scale, BAP-65, and CFS) will be evaluated using correlation coefficients (Pearson or Spearman, as appropriate). Due to the high heterogeneity of patients with AECOPD in the ED, multivariable analyses will be performed to account for potential confounding factors. Regression models will be adjusted for specific clinical covariates, including demographic characteristics, relevant comorbidities, baseline disease severity indicators, and validated clinical severity scores (SOFA, DECAF, BAP-65, Ottawa COPD Risk Scale, and Clinical Frailty Scale). The number of predictors included in multivariable models will be limited in relation to the number of observed outcome events (approximately 5–10 events per predictor when possible). Effect sizes and confidence intervals will be reported alongside *p*-values to facilitate appropriate interpretation of results. Receiver Operating Characteristic (ROC) curve analysis will be applied to determine the discriminative performance of individual biomarkers, and the area under the curve (AUC) will be calculated to identify optimal cutoff values associated with adverse outcomes. Short-term prognosis will be further analyzed through Kaplan–Meier survival curves and log-rank tests for 28-day mortality. A two-tailed *p*-value < 0.05 will be considered statistically significant for all analyses.

## 3. Expected Results and Anticipated Discussion

In this study, we expect to identify a composite biomarker profile-combining resistin, CC16, IL-6, TNF-α, and S100β-that improves early prognostic assessment in AECOPD beyond established clinical scores and routine laboratory indicators. It is important to emphasize that biomarker measurements obtained at ED admission are used exclusively as early prognostic indicators. Their purpose is to examine whether higher baseline levels predict progression to supplemental oxygen, NIV, or IMV, without influencing therapeutic decisions during hospitalization.

Three pre-specified findings are anticipated:

- Early biomarker–outcome correlations.

Higher admission levels of inflammation and injury biomarkers are expected to correlate with the need for ventilatory escalation, prolonged hospital stay, and increased 28-day mortality. Prior studies demonstrated that pro-inflammatory cytokines-particularly IL-6 and TNF-α- rise during acute exacerbations and are associated with clinical deterioration, frequent relapses, and mortality, supporting their role as prognostic markers in acute care models [14,15].

In COPD cohorts, resistin levels increase during exacerbation and decline upon recovery, mirroring systemic inflammatory burden and multi-organ stress [4,5,10,11]. These dynamics suggest its potential as a broad indicator of systemic inflammation in AECOPD [6].

II. Added value over clinical scores.

We expect incremental prognostic accuracy when integrating biomarker panels with validated Emergency Department risk tools such as DECAF [7], Ottawa COPD Risk Scale [9], and BAP-65 [8]. This integration could enhance discrimination and calibration for early triage decisions (e.g., progressive escalation from oxygen therapy to non-invasive ventilation, and ultimately invasive mechanical ventilation) and inform safer de-escalation strategies. While these scores already predict short-term outcomes effectively, adding an objective biological layer may refine their real-time predictive performance and improve individualization of care in the ED.

III. Mechanistic read-outs with management implications.

Lower CC16, a marker of bronchiolar epithelial integrity, has been causally associated with increased COPD susceptibility and faster progression in Mendelian randomization analyses [6]. In the acute setting, reduced CC16 concentrations may reflect extensive epithelial damage and predict more severe trajectories in AECOPD [12,13].

In parallel, S100β may index hypoxic neuro-injury during severe exacerbations. Elevated serum S100β has been linked to cerebral ischemic stress and neuronal damage in acute respiratory and critical illnesses, supporting its potential role as a neuro-systemic prognostic adjunct in the ED [16,17,22,23].

Originality and downstream impact:

To our knowledge, no ED based study has jointly evaluated the prognostic value of resistin, CC16, IL-6, TNF-α, and S100β in patients with AECOPD. Prior studies have assessed these molecules individually or in non-acute contexts, but none have integrated them within a unified, outcome-oriented framework [4,5,6,7,8,9,10,11,12,13,14]. Our study addresses this gap and is designed to derive cut-off thresholds and a parsimonious composite prognostic score applicable to emergency workflows.

If confirmed, these findings will support biomarker-guided algorithms—for instance, thresholds triggering NIV/IMV escalation or identifying extubation readiness when combined with diaphragmatic ultrasound—and may inform future therapeutic trials aimed at modulating inflammatory and epithelial-repair pathways (e.g., anti-TNF agents or CC16-protective interventions).

## Figures and Tables

**Table 1:** Objectives proposed by the research team.

Primary Objective	Secondary Objectives
To evaluate the association between serum levels of resistin, CC16, IL-6, TNF-α, and S100β measured at ED admission and early outcomes, including discharge status and 28-day survival, in patients presenting with acute exacerbations of COPD (AECOPD).	To determine biomarker cutoff values associated with the need for escalation of respiratory support (oxygen therapy, non-invasive ventilation, or invasive mechanical ventilation).
	To identify combinations of circulating biomarkers and established clinical scores that best predict short-term mortality and organ dysfunction.
	To assess the relationship between diaphragmatic contractility evaluated by bedside ultrasound and circulating biomarker levels, in relation to the level and duration of ventilatory support.
	To explore the potential role of biomarker-based risk stratification in informing clinical decision-making for AECOPD patients in the ED.

Legend: CC16—club cell secretory protein 16; IL-6—interleukin-6; TNF-α—tumor necrosis factor-alpha; S100β—S100 calcium-binding protein beta; ED—Emergency Department; COPD—chronic obstructive pulmonary disease; AECOPD—acute exacerbation of chronic obstructive pulmonary disease.

**Table 2:** Inclusion criteria for eligible study participants.

Inclusion Criteria	
Age ≥ 50 years. Confirmed prior diagnosis of COPD, regardless of disease stage or ongoing therapy:	
(a) the GOLD spirometric grades	GOLD I (FEV_1_ ≥ 80%) GOLD II (50% ≤ FEV_1_ < 80%) GOLD III (30% ≤ FEV_1_ < 50%) GOLD IV (FEV_1_ < 30%)
(b) ABE category assessment	A—low symptom burden and low risk of exacerbations; 0–1 moderate exacerbations in the previous year, with no hospitalizations B—increased symptoms but low risk of exacerbations; 0–1 moderate exacerbations in the previous year, without hospitalization E—high exacerbation risk, defined as ≥ 2 moderate exacerbations or ≥1 exacerbation requiring hospitalization in the previous year, irrespective of the level of symptoms
Admission to the ED with acute exacerbation of COPD. Signed informed consent obtained from the patient or legal representative.	

Legend: COPD—chronic obstructive pulmonary disease; GOLD—Global Initiative for Chronic Obstructive Lung Disease; FEV_1_—forced expiratory volume in 1 s; ED—Emergency Department.

**Table 3:** Exclusion criteria for eligible study participants.

Exclusion Criteria	
Age < 50 years. Coexisting acute or chronic conditions that could influence respiratory failure:	
(a) respiratory infections without underlying COPD	pneumonia, acute bronchitis, acute pulmonary viral infections (influenza, RSV, SARS-CoV-2, etc.)
(b) respiratory non-COPD causes	pneumothorax, asthma, bronchiectasis exacerbation in the absence of COPD), cystic fibrosis, upper airway obstruction (tumor, foreign body, laryngeal edema), hyperventilation syndrome or panic attack
(c) acute exacerbation of pre-existing interstitial lung disease (ILD)	idiopathic pulmonary fibrosis, interstitial pneumonias associated with connective tissue diseases, sarcoidosis, hypersensitivity pneumonitis, drug- or radiation-associated ILD
(d) cardiac causes	acute heart failure, acute coronary syndromes, cardiac tamponade, clinically significant arrhythmias, cardiogenic shock
(e) thromboembolic disease	pulmonary embolism
(f) metabolic or systemic causes	metabolic acidosis (DKA, renal failure), severe anemia, sepsis from non-pulmonary sources, hypovolemic shock, anaphylactic shock
(g) trauma	burns or trauma of any localization
(h) neurological conditions affecting respiratory function	acute stroke, neuromuscular diseases causing respiratory depression (myasthenia gravis, Guillain-Barré syndrome, amyotrophic lateral sclerosis)
Pregnancy. Cardiac arrest prior to or during ED admission. Terminal—stage neoplastic disease. Patients under custodial measures or unable to provide consent. Failure to obtain informed consent.	

Legend: COPD—chronic obstructive pulmonary disease; RSV—respiratory syncytial virus; SARS-CoV-2—severe acute respiratory syndrome coronavirus 2; ILD—interstitial lung disease; DKA—diabetic ketoacidosis; ED—Emergency Department.

**Table 4:** Analytical Performance and Reference Values of the Selected Biomarkers.

Biomarker	ELISA Kit (Elabscience®, Biotechnology Inc., Wuhan, Hubei, China)	Detection Range (Units)	LOD/Sensitivity (Units)	CV% (Intra/Inter)	Specificity/Cross-Reactivity	Normal Values (Adults)
Resistin	Human Resistin ELISA Kit (E-EL-H1213)	0.31–20 ng/mL	<0.19 ng/mL	<10%/<12%	High analytical specificity; no detectable cross-reactivity with other adipokines	3–12 ng/mL; higher in smokers and older adults
CC16	Human CC16 ELISA Kit (E-EL-H6083)	0.78–50 ng/mL	<0.47 ng/mL	<10%/<12%	No detectable cross-reactivity with other secretoglobins (SCGB family)	4–10 ng/mL in non-smokers; up to 15 ng/mL in smokers
IL-6	Human IL-6 ELISA Kit (E-EL-H6156)	7.81–500 pg/mL	<4.69 pg/mL	<10%/<12%	Minimal cross-reactivity; no measurable interference with IL-1β, IL-8, TNF-α, IFN-γ	<5–7 pg/mL; age-related increase possible
TNF-α	Human TNF-α ELISA Kit (E-EL-H0109)	15.6–1000 pg/mL	<9.38 pg/mL	<10%/<12%	High specificity; no detectable cross-reactivity with TNF-β or related cytokines	<8–10 pg/mL
S100B	Human S100B ELISA Kit (E-EL-H1297)	15.6–1000 pg/mL	<9.38 pg/mL	<10%/<12%	No measurable cross-reactivity with S100A1, S100A8, or other calcium-binding proteins	<90–100 pg/mL in healthy adults

Legend: ELISA—enzyme-linked immunosorbent assay; LOD—limit of detection; CV—coefficient of variation; ng—nanogram; CC16—club cell secretory protein 16; SCGB—secretoglobin protein family; IL-6—interleukin-6; pg—pictogram; IFN-γ—interferon gamma; TNF-α—tumor necrosis factor-α. Analytical specificity is reported as provided by the manufacturer, indicating absence of detectable cross-reactivity with structurally related proteins.

## Data Availability

No new data were created or analyzed in this study. Data sharing is not applicable to this article.

## Abbreviations

The following abbreviations are used in this manuscript:

AECOPD	Acute Exacerbation of Chronic Obstructive Pulmonary Disease
ALT	Alanine Aminotransferase
AST	Aspartate Aminotransferase
BAP-65	Blood urea nitrogen, Altered mental status, Pulse ≥ 109/min, Age ≥ 65 years score
CC16	Club Cell Secretory Protein 16
CFS	Clinical Frailty Scale
CO_2_	Carbon Dioxide
COPD	Chronic Obstructive Pulmonary Disease
CRP	C-Reactive Protein
CT	Computed Tomography
CV	Coefficient of Variation
DBP	Diastolic Blood Pressure
DECAF	Dyspnea, Eosinopenia, Consolidation, Acidemia, Atrial Fibrillation score
DKA	Diabetic Ketoacidosis
ED	Emergency Department
ELISA	Enzyme-Linked Immunosorbent Assay
EtCO_2_	End-Tidal Carbon Dioxide
EU	European Union
FEV_1_	Forced Expiratory Volume in 1 Second
FiO_2_	Fraction of Inspired Oxygen
GCS	Glasgow Coma Scale
GDPR	General Data Protection Regulation
GOLD	Global Initiative for Chronic Obstructive Lung Disease
HCO_3_^−^	Bicarbonate
hs-cTnI	High-Sensitivity Cardiac Troponin I
IFN-γ	Interferon Gamma
IL-6	Interleukin-6
ILD	Interstitial Lung Disease
IMV	Invasive Mechanical Ventilation
IQR	Interquartile Range
LOD	Limit of Detection
MAP	Mean Arterial Pressure
mMRC	Modified Medical Research Council Dyspnea Scale
NIV	Non-Invasive Ventilation
NT-proBNP	N-terminal pro–B-type Natriuretic Peptide
PaCO_2_	Partial Pressure of Arterial Carbon Dioxide
PaO_2_	Partial Pressure of Arterial Oxygen
pH	Potential of Hydrogen
ROC	Receiver Operating Characteristic
RSV	Respiratory Syncytial Virus
SBP	Systolic Blood Pressure
SARS-CoV-2	Severe Acute Respiratory Syndrome Coronavirus 2
SCGB	Secretoglobin Protein Family
SD	Standard Deviation
S100β	S100 Calcium-Binding Protein Beta
SOFA	Sequential (Sepsis-Related) Organ Failure Assessment
SpO_2_	Peripheral Oxygen Saturation
T°C	Core Body Temperature (degrees Celsius)
TNF-α	Tumor Necrosis Factor Alpha

## References

[1] Global Burden of Disease Study 2013 Collaborators (2015). Global, regional, and national incidence, prevalence, and years lived with disability for 301 acute and chronic diseases and injuries in 188 countries, 1990–2013: A systematic analysis for the Global Burden of Disease Study 2013. Lancet.

[2] Salvi S.S., Barnes P.J. (2009). Chronic obstructive pulmonary disease in non-smokers. Lancet.

[3] Agustí A., Celli B.R., Criner G.J., Halpin D., Anzueto A., Barnes P., Bourbeau J., Han M.K., Martinez F.J., Montes de Oca M. (2023). Global Initiative for Chronic Obstructive Lung Disease 2023 Report: GOLD Executive Summary. Am. J. Respir. Crit. Care Med..

[4] Vassiliou A.G., Vitsas V., Kardara M., Keskinidou C., Michalopoulou P., Rovina N., Dimopoulou I., Orfanos S.E., Tsoukalas G., Koutsoukou A. (2020). Study of inflammatory biomarkers in COPD and asthma exacerbations. Adv. Respir. Med..

[5] Che L., Xie Z., Chen G., Zhang W., Xia T., Lin J., Luo W., Chen L., Yin W., Cai X. (2023). The Mechanisms of Resistin-Like Molecule-β-Mediated Airway Inflammation in Chronic Obstructive Pulmonary Disease via Autophagy. J. Inflamm. Res..

[6] Milne S., Li X., Hernandez Cordero A.I., Yang C.X., Cho M.H., Beaty T.H., Ruczinski I., Hansel N.N., Bossé Y., Brandsma C.A. (2020). Protective effect of club cell secretory protein (CC-16) on COPD risk and progression: A Mendelian randomisation study. Thorax.

[7] Steer J., Gibson J., Bourke S.C. (2012). The DECAF Score: Predicting hospital mortality in exacerbations of chronic obstructive pulmonary disease. Thorax.

[8] Shorr A.F., Sun X., Johannes R.S., Yaitanes A., Tabak Y.P. (2011). Validation of a novel risk score for severity of illness in acute exacerbations of COPD. Chest.

[9] Stiell I.G., Perry J.J., Clement C.M., Brison R.J., Rowe B.H., Aaron S.D., McRae A.D., Borgundvaag B., Calder L.A., Forster A.J. (2018). Clinical validation of a risk scale for serious outcomes among patients with chronic obstructive pulmonary disease managed in the emergency department. CMAJ.

[10] Tat R.M., Golea A., Rahaian R., Vesa Ş.C., Ionescu D. (2019). Resistin and Cardiac Arrest-A Prospective Study. J. Clin. Med..

[11] Bonaventura A., Carbone F., Vecchié A., Meessen J., Ferraris S., Beck E., Keim R., Minetti S., Elia E., Ferrara D. (2020). The role of resistin and myeloperoxidase in severe sepsis and septic shock: Results from the ALBIOS trial. Eur. J. Clin. Investig..

[12] Kropski J.A., Fremont R.D., Calfee C.S., Ware L.B. (2009). Clara cell protein (CC16), a marker of lung epithelial injury, is decreased in plasma and pulmonary edema fluid from patients with acute lung injury. Chest.

[13] Hu X., Xu J., Li P., Zheng H. (2023). Correlation of Serum Clara Cell Secretory Protein 16, Plasma Fibrinogen and Serum Amyloid A with the Severity of Acute Exacerbated COPD and Their Combination in Prognosis Assessment. Int. J. Chronic Obstr. Pulm. Dis..

[14] Yao Y., Zhou J., Diao X., Wang S. (2019). Association between tumor necrosis factor-α and chronic obstructive pulmonary disease: A systematic review and meta-analysis. Ther. Adv. Respir. Dis..

[15] Hlapčić I., Belamarić D., Bosnar M., Kifer D., Vukić Dugac A., Rumora L. (2020). Combination of Systemic Inflammatory Biomarkers in Assessment of Chronic Obstructive Pulmonary Disease: Diagnostic Performance and Identification of Networks and Clusters. Diagnostics.

[16] Abboud T., Rohde V., Mielke D. (2023). Mini review: Current status and perspective of S100B protein as a biomarker in daily clinical practice for diagnosis and prognosticating of clinical outcome in patients with neurological diseases with focus on acute brain injury. BMC Neurosci..

[17] Tat R.M., Golea A., Vesa Ş.C., Ionescu D. (2019). Determination of Cut-off Serum Values for Resistin and S100B Protein in Patients Who Survived a Cardiac Arrest. J. Crit. Care Med..

[18] Celli B.R., Fabbri L.M., Aaron S.D., Agusti A., Brook R., Criner G.J., Franssen F.M.E., Humbert M., Hurst J.R., O’Donnell D. (2021). An Updated Definition and Severity Classification of Chronic Obstructive Pulmonary Disease Exacerbations: The Rome Proposal. Am. J. Respir. Crit. Care Med..

[19] Celli B.R., Fabbri L.M., Criner G.J., Papi A., Torres A., Agusti A. (2025). The ROME COPD Exacerbation Proposal Works! Time to Move Forward. Am. J. Respir. Crit. Care Med..

[20] Evans L., Rhodes A., Alhazzani W., Antonelli M., Coopersmith C.M., French C., Machado F.R., Mcintyre L., Ostermann M., Prescott H.C. (2021). Surviving Sepsis Campaign: International Guidelines for Management of Sepsis and Septic Shock 2021. Crit. Care Med..

[21] Martin-Loeches I., Torres A., Nagavci B., Aliberti S., Antonelli M., Bassetti M., Bos L.D., Chalmers J.D., Derde L., de Waele J. (2023). ERS/ESICM/ESCMID/ALAT guidelines for the management of severe community-acquired pneumonia. Intensive Care Med..

[22] Jalali R., Godlewska I., Fadrowska-Szleper M., Pypkowska A., Kern A., Bil J., Manta J., Romaszko J. (2023). Significance of S100B Protein as a Rapid Diagnostic Tool in Emergency Departments for Traumatic Brain Injury Patients. J. Pers. Med..

[23] Goyal A., Failla M.D., Niyonkuru C., Amin K., Fabio A., Berger R.P., Wagner A.K. (2013). S100b as a prognostic biomarker in outcome prediction for patients with severe traumatic brain injury. J. Neurotrauma.

